# Sex differences in gut fermentation and immune parameters in rats fed an oligofructose-supplemented diet

**DOI:** 10.1186/s13293-015-0031-0

**Published:** 2015-08-06

**Authors:** Padmaja Shastri, Justin McCarville, Martin Kalmokoff, Stephen P.J. Brooks, Julia M. Green-Johnson

**Affiliations:** Applied Bioscience Graduate Program and Faculty of Science, University of Ontario Institute of Technology, 2000 Simcoe Street North, Oshawa, Ontario L1H 7K4 Canada; Atlantic Food and Horticulture Research Center, Agriculture and Agri-Food Canada, Kentville, Nova Scotia B4N 1J5 Canada; Bureau of Nutritional Sciences, Health Canada, Ottawa, Ontario K1A 0K9 Canada

**Keywords:** Sex, Oligofructose, Microbiota, IgA, Liver, Butyrate

## Abstract

**Background:**

Mechanistic data to support health claims is often generated using rodent models, and the influence of prebiotic supplementation has largely been evaluated using male rodents. Given that sex-based differences in immune parameters are well recognized and recent evidence suggests differences in microbiota composition between sexes, validation of the effectiveness of prebiotics merits assessment in both males and females. Here, we have compared the effect of oligofructose (OF) supplementation on the fecal bacterial community, short chain fatty acid profiles, and gut mucosal and systemic immune parameters in male and female rats.

**Methods:**

Male and female rats were fed rodent chow or chow supplemented with OF (5 % *w*/*w*). Fecal community change was examined by analyzing 16S rRNA gene content. To compare effects of OF between sexes at the gut microbial and mucosal immune level, fecal short chain fatty acid and tissue cytokine profiles were measured. Serum lipopolysaccharide levels were also evaluated by the limulus amebocyte lysate assay as an indirect means of determining gut permeability between sexes.

**Results:**

In the fecal community of females, OF supplementation altered community structure by increasing abundance in the Phylum *Bacteroidetes*. In male rats, no changes in fecal community structure were observed, although fecal butyrate levels significantly increased. Liver Immunoglobulin A (IgA) levels were higher in males relative to females fed OF, and serum LPS concentrations were higher in males independent of diet. Females had higher basal levels of the regulatory cytokine interleukin-10 (IL-10) in the colon and liver, while males had higher basal levels of the pro-inflammatory cytokines IL-6 and cytokine-induced neutrophil chemoattractant-1 (CINC-1) in the cecum and liver.

**Conclusions:**

We have shown that male and female rat gut communities metabolize an OF-supplemented diet differently. Sex-specific responses in both the fecal community and systemic immune parameters suggest that this difference may result from an increase in the availability of gut peptidyl-nitrogen in the males. These findings demonstrate the importance of performing sex-comparative studies when investigating potential health effects of prebiotics using rodent models.

## Background

Scientific validation of health claims is required by regulators to ensure that they are accurate and do not mislead the public. For prebiotics, substantiation not only requires demonstration of a health benefit associated with consumption but also that selective stimulation of a specific genus or group of gut bacteria and/or a metabolic activity linked with these bacteria is associated with this health improvement [1]. In health claim submissions, supporting evidence often includes animal studies. These typically involve rodents and are of particular significance in mechanistic studies [2]. However, in order to avoid confounding effects from the estrus cycle, these are typically carried out using males only, limiting insight into potential sex differences. Sexual dimorphism in immune parameters is well documented [3]. Recently, sex-based differences in certain genes associated with histone-modifying activity, and of genes associated with control of infection and inflammation, were observed in the small intestine and colon of prepubescent C57BL/6 mice [4], illustrating sexual dimorphism at the level of mucosal immunity. Evidence also supports sex-associated differences in gut community composition [5–7] and gut community metabolic activity [8]. Sex-dependent effects of diet on the gut microbiota have also recently been demonstrated [9]. While the mechanisms underlying interactions between sex, microbiota, and diet have yet to be fully elucidated, these differences demonstrate potential limitations in extrapolating from studies of effects of dietary prebiotics on males to effects on females.

Fructans are found in a wide variety of cereals, fruits, and vegetables, where they function as plant storage polymers [10]. In North America, estimated dietary intake ranges from 1 to 4 g/day, somewhat lower than in Europe where intakes range from 3 to 11 g/day [11]. Fructans are composed of fructose polymers linked by β1-2 glycosidic bonds that often terminate in a glucose molecule and differ in length depending on the source (plant material vs. *de novo* synthesis) and method of processing [12]. In general terms, fructans fall within three classes; inulin (>10 degrees of polymerization (dp)), oligofructose (OF; ≤10 dp), and fructooligosaccharides (FOS; 1–5 dp) [10, 13]. Human and rat digestive enzymes are unable to cleave the β1-2 glycosidic bond, so fructans pass intact through the small intestine into the colon where they are digested by the gut bacterial community. These compounds are often referred to as prebiotics, and increased dietary intakes are claimed to improve host gut health by selectively stimulating the growth of bifidobacteria [14]. In humans though, the extent of this specific change can vary from person to person [15]. In rats, fructans appear to stimulate the growth of bifidobacteria, lactobacilli, and other lactic acid bacteria [16–18], although growth stimulation of other taxa including obligate anaerobes may also occur [19–21]. Fructans have been observed to increase the proportion of the short chain fatty acid (SCFA) butyrate in the gut [22–25]. Butyrate not only represents the primary energy source for colonocytes but is also thought to play an important role in the maintenance of gut health [26].

Feeding fructan-supplemented diets has previously been shown to affect gut barrier function. Feeding male rats FOS or inulin-supplemented diets increases Salmonella translocation [27, 28], gut permeability, and mucin sloughing [29, 30]. The latter effect has been observed in one study of human male subjects fed FOS [31] but not in another [32]. Adverse impacts on the gut following the ingestion of fructans are thought to result from their very rapid fermentation, resulting in abnormally high localized concentrations of SCFA (and possible low pH) in the proximal colon or cecum leading to epithelial injury [27, 33].

In addition to physiological effects, fructan feeding can also affect the immune system at the gut mucosal level. For example, inulin or FOS consumption can increase lymphocyte numbers in the Peyer’s patches of female rodents under healthy and endotoxemic conditions, indicating an impact on the main inductive portion of gut-associated lymphoid tissue (GALT) in the small intestine [34, 35]. FOS intake has also been noted to up-regulate secretion of Immunoglobulin A (IgA), a mucosal marker of gut homeostasis, in cecal and fecal contents of rodents [36–38]. Mucosal IgA production is in turn influenced by multiple cytokines, including interleukin-4 (IL-4), transforming growth factor β (TGF-β), IL-10, and IL-6, that also regulate the activity of other immune cells of the GALT [39], and production of certain of these cytokines has been reported to be influenced by fructan intake [36, 38]. Fructan-mediated effects on the immune system may reflect actions of SCFA produced through fermentation and also potentially direct effects mediated through interactions with toll-like receptors (TLR) on intestinal epithelial cells (IEC) and other immune cells, including monocytes [40–42].

Fructan ingestion can also affect systemic immune parameters at the level of blood and spleen [35, 43, 44], although relatively few studies have examined effects at the level of systemic immunity. Impacts at the gut mucosal level would also be anticipated to induce systemic changes via the gut–liver axis. Kupffer cells (liver macrophages), hepatocytes, and endothelial cells help maintain immune regulation at the liver by inducing tolerance to gut-derived antigens [45, 46]. The liver is one of the tissues that is sexually dimorphic in its gene expression [47, 48], potentially contributing to sex-associated effects of fructan-containing diets on systemic immune markers.

Here, we have examined how a diet supplemented with OF influences the metabolic activity and structure of the fecal bacterial community and systemic host-immune parameters in both female and male rats. Sex-associated differences in the metabolism of this diet illustrate the importance of including both males and females in animal studies used to investigate the universality of health effects of dietary fibers and fermentable substrates.

## Methods

### Feeding trial

Forty eight, 28–42-day-old, BioBreeding control male (*n* = 24) and female rats (*n* = 24) were obtained from the Animal Resource Division of Health Canada. The BioBreeding control rat is an in-house bred rat line originally derived from Wistar rats. Rats were fed a control diet (Purina 5001 rodent chow, 12 males and 12 females) or rodent chow supplemented with 5 % (*w*/*w*) OF (dp 2–8, Orafti, Pemuco, Chile; 12 males and 12 females) for 42 days. Rats had free access to reverse-osmosis treated water, were separately housed within individual mesh bottomed-stainless steel cages, and subjected to a 12-h light/dark cycle. A 2-week acclimatization period (control diet) preceded the 6-week experimental feeding trial, after which the rats were euthanized and tissues were collected. This study was approved by the Health Canada Animal Care Committee and the University of Ontario Institute of Technology Animal Care Committee (AUP 12–003). Six animals from each treatment group were randomly selected for cytokine analysis, and the remaining six used for immunophenotyping and fecal community analysis.

### Analysis of fecal SCFAs

Gas chromatographic analysis of fecal SCFAs and branch-chain fatty acids (BCFAs) were performed as previously reported [49].

### Isolation of fecal DNA, construction and analysis of 16S rRNA gene libraries, and real-time (q)PCR

Fecal pellets were collected from each rat at necroscopy. Feces from individual rats were sampled by weight (1.0 g/rat) and then pooled based on diet and sex (*n* = 6 pellets × four treatment groups). Samples were pooled to reduce inter-rat fecal community variability [50]. Pooled feces from each treatment group were frozen in liquid nitrogen, ground to a fine powder using a mortar and pestle, and duplicate community DNA samples isolated using the QIAamp DNA stool Mini Kit (Qiagen Inc., Mississauga, Ontario). DNA was stored frozen at −20 °C. Duplicate near full length 16S rRNA gene libraries were prepared from the DNA isolated from each treatment group. Libraries were constructed as previously described using primers F44 and R1592 [51], with amplicons cloned into the vector pCR2.1-TOPO and transformed into *Escherichia coli* TOP10 (Invitrogen, Carlsbad, California). One hundred transformants were randomly picked from each duplicate clone library, the plasmids isolated and sequenced as previously described [51]. Sequence alignments were carried out against the Silva data base [52] and checked for chimeric sequences using Chimera-slayer, all implemented through Mothur [53]. Sequences were initially binned based on their best match in the Silva data base [52] <3 % sequence divergence) followed by a ClustalW alignment and the generation of nearest neighbor trees [54] with sequences aligning ≤3 % sequence divergence further binned. Phylotypes were classified using Seqmatch [55]. Specific changes in 16S rRNA gene content in fecal DNA under each treatment were also determined by quantitative (q)-PCR using previously described oligonucleotide primer sets and conditions for quantification of the phylum *Bacteroidetes* [56] and the genus *Lactobacillus* [57]. Gene copy numbers (copies/ng community DNA) were expressed as a percentage of the total bacterial 16S rRNA gene content determined using the universal primers HDA1 and HDA2 and conditions as previously described [51].

### Tissue preparation

Blood was collected during necroscopy from the portal vein of the liver. GI tract tissues (ileum, cecum, and colon) and systemic tissues (liver) were collected, snap frozen, and stored at −80 °C and subsequently homogenized in the presence of a protease inhibitor cocktail (Sigma Chemical Co.) as previously described [58]. Homogenized tissues were centrifuged for 30 min at 16,100×*g*. Supernatants were then recovered and stored at −80 °C for further cytokine and immunoglobulin analysis.

### Serum biochemistry

Individual serum samples were analyzed for blood urea nitrogen (BUN) and lipopolysaccharide (LPS) content. BUN levels were measured using the urease-glutamate dehydrogenase method [59] using an ABX Pentra 400 Automated clinical chemistry analyzer and ABX Pentra test kits for BUN (Horiba Canada Inc., Burlington, ON, Canada). Serum endotoxin (LPS) concentrations were analyzed using the endpoint chromogenic limulus amebocyte lysate (LAL) assay (Lonza, Switzerland). The assay was performed according to the manufacturer’s protocol and measurable levels of LPS were expressed as EU/ml.

### Flow cytometry

Flow cytometric analysis of mucosal (mesenteric lymph node) and systemic (spleen) samples was performed using a Becton Dickinson fluorescent activated cell sorter (FACSCalibur flow cytometer, San Jose, California). Gating techniques were as previously described [60]. Antibodies used for cell surface staining were anti-CD45 (PE-Cy5, Clone OX-1; leukocyte common antigen), anti-CD3 (APC, Clone 1 F4; total T cell), anti-CD4 (PE, Clone OX-35; T helper), anti-CD8a (FITC, Clone OX-8; cytotoxic T cell), anti-CD45RA (FITC, Clone OX-33; B cell), and anti-CD161a (PE, Clone 10/78; natural killer cell). Intensity of macrophage (CD68) expression was also analyzed as an indicator of macrophage activation using anti-CD68 (Alexa Fluor 488, ED1 clone) [61]. Fluorochrome-matched isotype controls were used to assess non-specific binding. All antibodies were obtained from BD Biosciences (Mississauga, Ontario), except for CD68 which was purchased from AbD Serotec (Raleigh, North Carolina).

### Cytokine and IgA analysis

Cytokines (CINC-1, IL-6, TGF-β1, IL-4, and IL-10) and immunoglobulin IgA concentrations were measured by enzyme-linked immunosorbent assays (ELISAs) using manufacturer’s protocols (R&D systems and Bethyl Laboratories, respectively). ELISAs were performed using streptavidin conjugated to horseradish-peroxidase, and plates were read at a wavelength of 450 nm on a Synergy HTTR microplate reader (Bio-Tek, USA). Results for cytokines and IgA were expressed as ng/g or μg/g of tissue, respectively.

### Statistical analysis

Cluster analysis of 16S rRNA gene libraries was carried out using PC-ORD (MjM Software, Gleneden Beach, Oregon) using a Bray-Curtis distance measure and the group average linkage method. Rarefaction and fecal community diversity indices were obtained using the Fastgroup II on line software [62]. Immunological data was analyzed using a two-way and three-way analysis of variance (ANOVA) to examine overall effects of diet, sex, and tissue, as appropriate. Differences were further analyzed using a multiple range test when ANOVA *p* < 0.050. Data were checked for a potential correlation between means and SD before ANOVA. When a correlation was observed, the data were transformed using the Box-Cox formula: *T*(*Y*) = (*Y*^lambda − 1)/lambda where *Y* is the response variable and lambda is the transformation parameter [63]. Values of lambda were chosen to minimize the mean-square error. Unless otherwise indicated, values represent means ± SEM for indicated number of rats.

## Results

### Feeding trial and fecal SCFA/BCFA

**Fig. 1:**
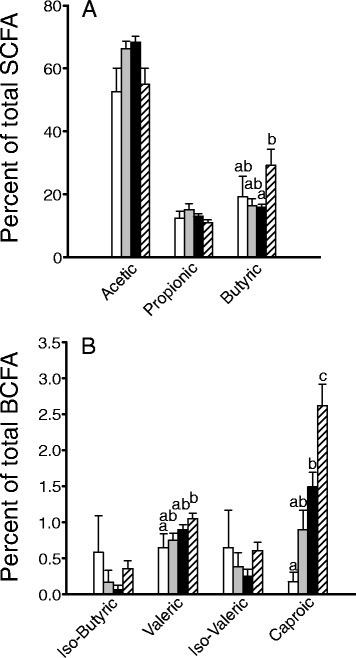
SCFA (**a**) and BCFA (**b**) output in rats fed rodent chow or rodent chow supplemented with OF. Data are shown as mean ± SEM (*n* = 6–8); significant differences in SCFA or BCFA concentrations are indicated by different *letters*. Columns are arranged as follows: females fed rodent chow, females fed chow supplemented with OF, males fed rodent chow, males fed chow supplemented with OF

Weight gain (Table 1) was unaffected by diet in males and females (*p* = 0.31) but differed significantly between sexes (*p* < 0.001). Energy consumption was also unaffected by diet (*p* = 0.57) but was higher in males compared to females (*p* < 0.001). Differences in food intake and energy intake reflected the final body weights at necropsy (*p* < 0.001). OF-fed male and female rats had significantly larger ceca (*p* < 0.003). Total fecal fatty acid outputs (Fig. 1) of rats fed OF (μmol day-1) were approximately 1.3× those fed chow (*n* = 6 males and *n* = 6 females per diet group; *p* = 0.002), although fecal outputs (g dry weight d-1) were not significantly different (*p* = 0.32). SCFA and BCFA profiles changed with both diet and sex. In females consuming chow, acetate levels were lower than in those consuming OF, although this difference was not significant. The OF-supplemented diet significantly increased butyrate and caproic acid outputs in males. Other BCFAs also trended upwards in males fed OF.

**Table 1:** Food intake and growth characteristics^a^

	Male		Female	
	Control	OF	Control	OF
Weight gain (g)	135.2 ± 3.5	140.2 ± 3.5	55. 9 ± 3.9^*^	55.6 ± 3.7^*^
Total weight^b^ (g)	392.0 ± 6.1	401.1 ± 3.9	227.5 ± 5.0^*^	231.0 ± 5.1^*^
Energy Intake (kcal/d)	93.0 ± 1.6	90.6 ± 1.1	71.6 ± 1.5^*^	76.1 ± 2.8^*^
Cecal weight (g)	7.6 ± 0.2	9.9 ± 0.2	5.1 ± 0.3^*^	6.5 ± 0.4^*^

*OF* oligofructose

*Significance based on sex as analyzed by ANOVA

^a^Values are presented as mean ± SEM for male (*n* = 13–18) and female (*n* = 12) rats fed the control or OF-supplemented diet

^b^The total weight was taken at necropsy

### Impact of oligofructan on the fecal bacterial community

**Fig. 2:**
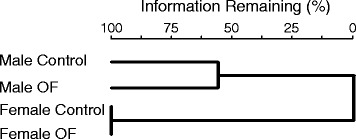
Cluster analysis comparing community diversity (phylotype occurrence and abundance) in feces from rats fed rodent chow or OF-supplemented rodent chow

**Fig. 3:**
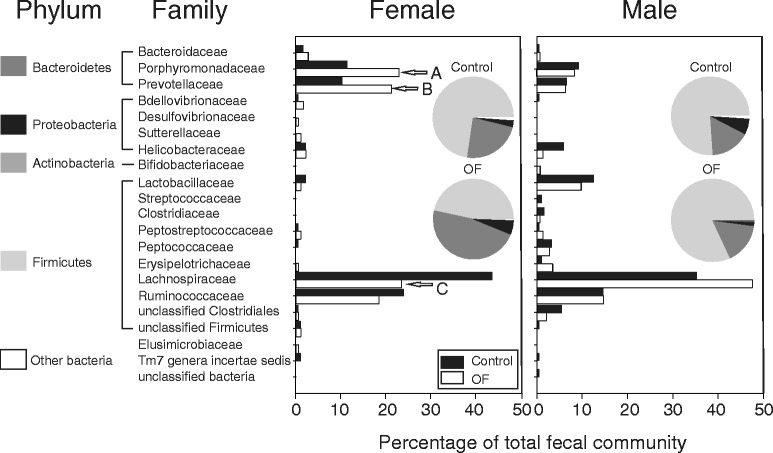
Change in the male and female fecal community in response to diet. *Bar graphs* indicate the distribution of phylotypes (%) at the family level. Rats fed rodent chow (*black bar*) or rodent chow supplemented with OF (*white bar*). *Left panel*: females. *Right panel*: males. *Arrows* indicate major taxa undergoing change in response to OF (*A*) Family *Porphyromonadaceae*. (*B*) Family *Prevotellaceae.* (*C*) Family *Lachnospiraceae. Inset*: Pie charts show the distribution of phylotypes at the level of phylum in rats fed rodent chow (*upper* pie charts) or rodent chow supplemented with OF (*lower* pie charts)

A total of 104 near full length 16S rRNA phylotypes were identified in the feces obtained from these rats, a level of community richness somewhat lower than is typically found by pyrosequencing (50, 64). Approximately 44 % of these were shared between the male and female control fecal communities, while only 25 % were shared among all four treatment groups. In females, 70 % of the phylotypes were shared between diets, compared to only 60 % in the males. Feeding OF reduced fecal community richness in males, increased it in females, but had little effect on fecal community diversity (Table 2). A cluster analysis indicated that the communities partitioned by sex rather than diet (Fig. 2). At the phylum level, fecal community structure was similar between the males and females fed the control diet (upper pie charts, Fig. 3). In males, *Lactobacillaceae* content was higher than found in females and this was independent of diet (~11 % in males versus ~2 % in females). Feeding OF increased the content of *Bacteroidetes* in females (23 % in control versus 47 % in OF-fed females), but not males (16.1 % in the control versus 16.3 % in OF-fed males; lower pie charts, Fig. 3). Increases in the content of *Bacteroidetes* in females fed OF resulted from increases in the content of phylotypes primarily aligning within the Families *Porphyromonadaceae* (Genus *Barnesiella*) and *Prevotellaceae* (Genus *Prevotella*). Most of the phylotypes eliciting increased abundance in response to OF supplementation in females were also present in males (Table 3). Feeding males OF increased the content of phylotypes aligning within the Family *Lachnospiraceae*, but decreased their content in females (bar charts, Fig. 3). Differences in the abundance of the *Bacteroidetes* and lactobacilli were also determined by qPCR analysis using phylum and genus-specific primer sets and each of the fecal DNA pools. In females, OF increased the abundance of the *Bacteroidetes* (35.5 % in control versus 52.2 % in OF-fed females) but had no effect in the males (37.8 % in control versus 36.4 % in OF-fed males). While 16S rRNA libraries underestimated the content of *Bacteroidetes* within each community, the trend remained the same. Estimates for lactobacilli abundance were similar between both approaches, being higher in males (8.7 % in control versus 8.8 % in OF-fed males), than in females (3.6 % in control versus 1.5 % in OF-fed females).

**Table 2:** Estimates of richness (Chao1) and diversity (Shannon index) in the fecal community of male and female rats fed chow or the OF-supplemented diet

Treatment	Chao1^a^	Shannon index^a^
Male chow	106	3.7
Male chow + OF	80	3.7
Female chow	87	3.4
Female chow + OF	108	3.6

*OF* oligofructose

^a^Calculated using the Fastgroup II online software [62]

**Table 3:** Changes in the abundance of phylotypes aligning within the Genera *Barnesiella* and *Prevotella* in rats fed rat chow or rat chow supplemented with OF

Clone	RDP relative	S_ab^a^	RDP classification	Phylotype abundance (percent of total community)			
				Male	+OF	Female	+OF
A15461	S001381749	0.96	*Barnesiella*	1.1	0.0	0.0	0.0
F19056	S000715072	0.98	*Barnesiella*	0.0	0.0	0.5	2.2
F19074	S001381499	1.00	*Barnesiella*	0.0	0.0	0.5	0.6
G19307	S001380091	1.00	*Barnesiella*	1.1	1.4	0.0	3.9
G19322	S001080987	1.00	*Barnesiella*	0.0	0.0	0.5	1.1
G19350	S001380841	0.99	*Barnesiella*	0.0	0.0	0.5	0.6
G20673	S000707322	0.98	*Barnesiella*	0.5	0.7	1.1	0.6
G20691	S001380274	0.95	*Barnesiella*	1.1	1.4	0.5	2.2
G20696	S001381099	0.97	*Barnesiella*	0.5	0.7	0.0	2.8
			Total *Barnesiella*	4.3	4.2	3.8	14.0
F20815	S001380751	0.96	*Prevotella*	3.3	4.2	7.7	12.4
G19310	S003238090	0.98	*Prevotella*	2.2	0.7	1.6	6.7
			Total *Prevotella*	5.4	4.9	9.3	19.1
Total increase in *Barnesiella* and *Prevotella*				9.8	9.1	13.1	33.1

*OF* oligofructose

^a^Similarity coefficient RDP Seqmatch

### IgA concentrations

**Fig. 4:**
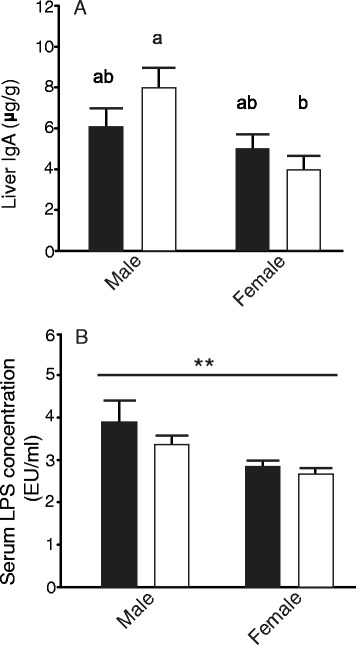
Changes in immune status in rats consuming rodent chow (*black bar*) or rodent chow supplemented with OF (*white bar*). **a** Liver IgA levels (μg/g) are shown as mean ± SEM (*n* = 5–6). Males demonstrated significantly (*p* = 0.01) elevated levels of liver IgA relative to females. An interaction (diet × sex; *p* = 0.04) for liver IgA was also detected by two-way ANOVA analysis of males compared to females consuming OF. Differing *letters* denote significant differences as determined by Tukey’s multiple range test. **b** Serum LPS concentrations (EU/ml) are shown as mean ± SEM (*n* = 4–5). **Significance (*p* = 0.004) based on sex, with males having elevated concentrations of serum LPS compared to females

Total IgA levels were determined in cecal contents and in liver tissue. Cecal IgA levels were unaffected by diet or sex (data not shown). In contrast, liver IgA concentrations in males were higher than in females, and increased significantly over females when fed OF (Fig. 4a).

### Serum biochemistry

In order to determine whether endogenous urea may have contributed to the gut fermentation in male and female rats fed either diet, we measured serum BUN levels. BUN levels were unaffected by diet or sex (males control/OF: 18.62 ± 0.9 and 17.94 ± 1.48, females control/OF: 18.94 ± 1.31 and 17.61 ± 0.92; *n* = 6). LPS concentrations in the serum of males and females fed either diet were determined to assess gut barrier functionality. Diet had no effect on serum LPS concentrations; however, males had significantly higher circulating LPS concentrations relative to females (Fig. 4b).

### GI tract and liver tissue cytokine changes

In order to examine immunological effects associated with OF, cytokine concentrations were measured in gastrointestinal tissues and liver. Basal cytokine levels varied significantly between tissues and with sex, with the exception of the regulatory cytokine TGF-β1 which was unaffected by sex (Table 4). In general, colonic tissue cytokine concentrations were higher in females than males. Although diet had no major effect on tissue cytokine levels, interactions for CINC-1 (diet × sex; *p* = 0.007) and for IL-6 (tissue × sex × diet; *p* = 0.02) were noted. At the systemic level, liver tissue concentrations of the pro-inflammatory cytokines IL-6 and CINC-1 were higher in males compared to females, while the concentration of the regulatory cytokine IL-10 was highest in the liver of females.

**Table 4:** Cytokine levels in gastrointestinal and liver tissue in male and female rats fed control or OF-supplemented diets

		Male		Female		Main and interaction (*p* value)					
Cytokine(ng/g)	Tissue^a^	Control	OF	Control	OF	Diet^b^	Sex^c^	Diet × sex^d^	Tissue × diet^e^	Tissue × sex^f^	Tissue × diet × sex^g^
CINC-1	Ileum	0.23 ± 0.01	0.25 ± 0.02	0.02 ± 0.02	0.17 ± 0.10						
	Cecum	0.89 ± 0.23	0.69 ± 0.14	0.14 ± 0.14	0.51 ± 0.19	0.08	**<** *0.001*	*0.007*	0.06	**<** *0.001*	NS
	Colon	0.70 ± 0.12	0.46 ± 0.07	1.59 ± 0.06	1.75 ± 0.13						
	Liver	10.95 ± 0.88	9.06 ± 0.96	6.56 ± 1.30	5.27 ± 0.38						
IL-6	Ileum	ND	ND	1.33 ± 0.45	2.41 ± 0.18						
	Cecum	7.85 ± 0.92	7.73 ± 0.86	5.83 ± 0.71	5.06 ± 0.54	NS	**<** *0.001*	NS	0.08	**<** *0.001*	*0.02*
	Colon	1.94 ± 0.24	2.45 ± 0.36	6.22 ± 1.17	4.59 ± 0.58						
	Liver	159.16 ± 11.94	173.42 ± 18.92	107.77 ± 9.43	93.81 ± 11.67						
TGF-β1	Ileum	0.47 ± 0.03	0.54 ± 0.04	ND	ND						
	Cecum	2.45 ± 0.91	2.22 ± 0.89	2.09 ± 1.04	2.50 ± 1.25	NS	NS	NS	NS	NS	NS
	Colon	0.44 ± 0.01	0.54 ± 0.03	0.72 ± 0.05	0.72 ± 0.04						
	Liver	36.97 ± 1.51	41.54 ± 2.24	39.36 ± 3.23	37.48 ± 4.70						
IL-4	Ileum	0.14 ± 0.02	0.13 ± 0.03	0.89 ± 0.16	1.12 ± 0.17						
	Cecum	2.92 ± 0.53	2.28 ± 0.37	1.98 ± 0.10	2.34 ± 0.07	NS	**<** *0.001*	0.08	NS	**<** *0.001*	NS
	Colon	1.06 ± 0.14	0.80 ± 0.07	3.85 ± 0.28	4.70 ± 0.12						
	Liver	32.56 ± 0.76	40.98 ± 6.13	48.99 ± 6.22	45.19 ± 6.49						
IL-10	Ileum	3.9 ± 0.30	4.92 ± 0.66	1.28 ± 0.2	1.24 ± 0.21						
	Cecum	5.30 ± 0.24	6.48 ± 1.27	8.16 ± 0.84	6.66 ± 0.34	NS	**<** *0.001*	NS	NS	**<** *0.001*	NS
	Colon	0.95 ± 0.13	0.77 ± 0.12	10.03 ± 0.68	9.84 ± 0.57						
	Liver	73.21 ± 4.18	75.39 ± 7.11	108.27 ± 11.09	111.42 ± 9.98						

*OF* oligofructose, *ND* not detectable, *NS* not significant

^a^Values denote the mean (ng/g) ± SEM (*n* = 3–6) for each tissue

^b^Significance for diet was detected using three-way ANOVA

^c^Significance for sex was detected using three-way ANOVA

^d^Interactions between diet × sex were detected using three-way ANOVA

^e^Interactions between tissue × diet were detected using three-way ANOVA

^f^Interactions between tissue × sex were detected using three-way ANOVA

^g^Interactions between tissue × diet × sex were detected using three-way ANOVA

### Mesenteric lymph node and spleen immune cell phenotyping

In the mesenteric lymph node (MLN), supplementing the diet with OF had no impact on the population distribution of immune cell phenotypes. However, significant differences were noted between immune cell phenotype distributions based on sex (Table 5). MLN total T cell (CD3^+^) percentages were significantly (*p* = 0.002) higher in females, while MLN total B cell (CD45RA^+^) percentages were significantly (*p* = 0.001) higher in males, irrespective of diet. Macrophage (CD68^+^) population percentages were higher in the MLN and spleen of females relative to males (*p* = 0.001; *p* = 0.002, respectively). Overall, diet significantly affected splenic total T cell (CD3^+^; *p* = 0.040) populations in females, with the lowest percentage of splenic T cells observed in rats consuming the OF-supplemented diet.

**Table 5:** Immune cell phenotypes in the mesenteric lymph node (MLN) and spleen of males and female rats consuming either the control rat chow or chow supplemented with OF

Tissue	Cell phenotype^a^	Male		Female		Diet^b^	Sex^c^	Diet × sex^d^
		Control	OF	Control	OF			
MLN	CD3^+^	72.62 ± 0.86	72.97 ± 1.47	76.98 ± 0.22	76.94 ± 1.25	NS	*0.002*	NS
	CD3^+^4^+^	52.19 ± 0.69	52.59 ± 0.73	54.79 ± 0.76	52.82 ± 1.43	NS	NS	NS
	CD3^+^8^+^	19.02 ± 1.22	18.31 ± 1.02	20.29 ± 0.67	21.83 ± 1.52	NS	NS	NS
	CD45RA^+^	23.36 ± 0.78	23.00 ± 1.55	18.80 ± 0.58	18.71 ± 1.25	NS	*0.001*	NS
	CD3^−^161^+^	0.33 ± 0.08	0.31 ± 0.08	0.39 ± 0.09	0.28 ± 0.04	NS	NS	NS
	CD68^+^	5.94 ± 0.77	5.49 ± 0.51	8.77 ± 0.78	8.09 ± 0.46	NS	*<0.001*	NS
Spleen	CD3^+^	71.67 ± 1.18	69.03 ± 1.96	73.03 ± 0.94	69.23 ± 1.12	*0.04*	NS	NS
	CD3^+^4^+^	45.53 ± 1.51	44.11 ± 1.43	46.10 ± 0.94	42.65 ± 1.21	NS	NS	NS
	CD3^+^8^+^	25.47 ± 0.90	24.93 ± 0.53	25.45 ± 0.67	25.04 ± 0.54	NS	NS	NS
	CD45RA^+^	18.52 ± 1.06	20.51 ± 1.39	15.41 ± 1.21	18.56 ± 1.43	NS	NS	NS
	CD3^+^161^−^	1.18 ± 0.2	1.57 ± 0.24	1.80 ± 0.18	1.38 ± 0.13	NS	NS	NS
	CD68^+^	41.58 ± 1.67	40.72 ± 1.49	45.97 ± 2.41	49.15 ± 1.28	NS	*0.002*	NS

*OF* oligofructose, *NS* not significant

^a^Cell phenotypes are expressed as the percent of the CD45^+^ (leukocyte common antigen) cell population. Values indicate the percent mean ± SEM (*n* = 5–6) for each immune cell type in the MLN and spleen

^b^Effects of diet were detected using two-way ANOVA

^c^Differences between sexes were detected using two-way ANOVA

^d^Significance of interactions was detected using two-way ANOVA

## Discussion

The gut communities in male and female rats metabolized an OF-supplemented chow differently, and this was reflected in changes to the relative proportions of fecal butyrate and certain BCFAs in males, and in differences in fecal community structure between the sexes. In contrast, when fed the control diet, both SCFA outputs and fecal community structures were very similar in both sexes. Where the control fecal communities differed was in terms of composition, sharing only 44 % of the identified phylotypes. Sex-based differences in the fecal communities of rodents have previously been observed [6, 65]. While OF supplementation affected fecal community composition in both male and female rats to varying extents, the resulting communities remained more similar based on sex rather than diet, and broad diet-associated changes in fecal community structure were limited exclusively to the females. Feeding OF had no impact on weight gain, but did significantly increase fecal SCFA outputs in both sexes. Owing to its high solubility, OF would be expected to be rapidly fermented in the cecum, consistent with the increased empty cecal weights observed here and in other studies [22].

In females, OF altered fecal community structure by increasing the content of phylotypes in the phylum *Bacteroidetes* (primarily the families *Prevotella* and *Barnesiella*), and this occurred largely at the expense of the family *Lachnospiraceae* (phylum *Firmicutes*). We have observed an identical shift in the feces of male rats fed various fermentable substrates, and this is thought to follow from increases in the availability of gut ammonia and its use as a nitrogen source to support continued microbial growth in the distal gut [64]. In fact, isolates in the genus *Prevotella* have a preference for ammonia as a nitrogen source [66] and we suspect that this is likely true for the yet to be cultivated species in the genus *Barnesiella*. Ammonia arises from the hydrolysis of endogenous urea and by the fermentation of dietary and endogenous proteins, peptides, and amino acids [67]. We would expect that gut urea concentrations in rats would generally be quite low, as has been observed in humans [68]. Furthermore, we found no indication of increased urea flux into the gut following OF supplementation (i.e., no change in blood urea nitrogen concentration) as observed in rats fed resistant starch [50]. On this basis, the bulk of the ammonia used to support microbial growth in the distal gut would likely originate from fermentation. Protein fermentation occurs concurrently with carbohydrate fermentation [50, 64] and ammonia resulting from deamination of the amino acids increases in concentration distally down the gut tract [69]. Increased nitrogen demand in the proximal gut due to the inclusion of an additional rapidly fermented substrate to the diet depletes the available peptidyl-nitrogen sources and in females resulted in a distal gut community where continued growth would be dependent primarily on ammonia. This change is reflected in an increase in the abundance of the phylum *Bacteroidetes* in the feces [64].

In contrast to females, fecal community structure in the OF-fed males remained similar to that found under the control diet. This finding is similar to a recent rat trial where feeding a different prebiotic (xylo-oligosaccharide) was also found to have quite modest impacts on the fecal community [70]. In males, OF significantly increased caproic acid output and an upward trend was also observed for the other BCFA concentrations. Since BCFAs are a marker for gut protein fermentation [69], it would be expected that increases in ammonia would also occur. It was therefore unusual that we observed no increase in the abundance of the *Bacteroidetes* in the fecal community of males, despite sharing many of the affected phylotypes with OF-fed females. Communities based on the analysis of 16S rRNA gene libraries are subject to a variety of artifacts which can potentially impact the inferred structure [71]. Indeed, direct determinations of *Bacteroidetes* content (qPCR) were generally higher than indicated by the clone libraries. We have previously observed similar differences in *Bacteroidetes* content in male rat fecal samples subjected to 16S rRNA pyrosequencing compared to determinations based on qPCR or shot gun metagenomics [50, 64]. However, despite this difference, the overall trend, i.e., the shift toward the *Bacteroidetes* in OF-fed females only, was identical between 16S rRNA and qPCR analysis. We suggest that sex-based differences in the fecal community response of rats fed OF might reflect differences in the availability of peptidyl-nitrogen in the distal gut.

Since male and female rats consumed a proportionally equivalent amount of protein types in both diets, either male rats have a reduced ability to digest dietary protein in the small intestine, allowing more protein to pass into the lower gut, or additional endogenous protein sources must be present in the male gut lumen, but absent in the females. Again, we did observe a trend toward higher relative concentrations of BCFAs in males fed OF which could indicate more protein fermentation than in the corresponding females. Moreover, results from our immunological analysis of these rats also support the latter. In Sprague–Dawley rats, the male colon is more permeable than that of females [72], and our results also support differences in gut barrier function independent of diet in male and female BioBreeding control rats. Firstly, serum LPS concentrations in male rats were higher than in females, independent of diet, a finding consistent with increased translocation of gut bacteria in males, as shown by others [27]. In addition, liver tissues from males showed significantly higher levels of the pro-inflammatory cytokines IL-6 and CINC-1. Both cytokines are involved in the immune response to inflammation and tissue injury, and increased IL-6 production is associated with mucosal barrier injury in male rats [72–74]. Finally, basal liver IgA levels were significantly higher in males than females and further increased when fed the OF-containing diet. IgA is the major immunoglobulin found in the mucosal secretions of mammals and is important in protecting the host from invasion by pathogenic bacteria [75]. In both rats and humans, liver macrophages (Kupffer cells) are involved in the removal of IgA-opsonized bacteria and antigens via FCRα1 receptors, an important role when gut barrier function is reduced [75, 76]. Although we did not directly analyze gut barrier integrity by methods such as FITC-dextran administration, these findings suggest a decrease in the gut barrier, leading to heightened pro-inflammatory cytokine and IgA production at the systemic level in male rats compared to females. A more permeable gut would in turn permit increased diffusion of host proteins into the lumen, providing a source of peptidyl-nitrogen for gut microbes.

Feeding fructans to rats is known to impact gut barrier function, inducing the shedding of mucin and increasing the translocation of gut pathogens [27–29]. While we observed systemic immune effects that would be consistent with heightened gut permeability in males, increased gut permeability in males fed OF might also result from a higher turnover of gut mucosal cells. Colonocyte maturation and differentiation appears to be dependent on butyrate [77, 78], which increased in fecal contents of males fed the OF-supplemented diet. A higher rate of colonic epithelial turnover would also increase protein availability in the male lumen and may further contribute to differences in gut permeability between males and females.

In contrast to the changes in liver pro-inflammatory cytokine levels associated with reduced gut barrier function in males, female liver and gastrointestinal tract tissue had high basal levels of IL-10, a cytokine known for its role in mucosal homeostasis [79]. Female rats consuming the OF-supplemented chow had the highest levels of IL-4 in the cecum and colon, tissues in which IL-10 levels were high. This is a cytokine profile indicative of T_H_2 activity, a T cell phenotype that supports antibody production and mediates immune homeostasis at the gut level [80, 81]. Additionally, females had a higher percentage of MLN CD68^+^ cells, indicating an increased presence of macrophages in the MLN along with higher levels of IL-10 compared to males. IL-4 and IL-10 are inducers of M2 phenotype macrophages that help maintain a tolerogenic gut environment and in turn differentiate T helper cells into T_H_2 [82]. These cell phenotypes and cytokine profiles would support greater gut barrier integrity [83] potentially contributing to differences between sexes in preventing gut-derived stimuli from entering into systemic compartments. Diet and sex also had differential effects at the systemic immune level. Splenic CD3^+^ (total T cell) percentages were affected by diet, with the lowest percentages observed in female rats consuming the OF-supplemented diet, while splenic macrophage (CD68^+^) populations were higher in females compared to males. Together, this suggests that immune population distributions can be selectively affected by diet and sex, at the systemic and mucosal level.

## Conclusions

We have examined the effects of feeding an OF-supplemented rodent chow diet on microbial metabolism and immune measures in male and female BioBreeding rats. Our results demonstrate that the gut community in male and female rats metabolized this diet differently, and this appears to result from a difference in the source of available fixed-nitrogen used to support gut microbial growth leading to differences in SCFA and BCFA profiles and downstream systemic immune effects between males and females. Animal studies represent an important tool to both investigate and provide evidence to support health claims for functional foods such as fructans, but traditionally, these studies have tended to avoid the use of female rodents. However, as demonstrated here, examining only male rats may lead to the erroneous conclusion that fructans are universally butyrogenic, while a complex metabolic and digestive interaction underlies this effect. Our findings not only demonstrate the importance of nitrogen source as a factor controlling community diversity in the lower gut of monogastric animals but also illustrate the need to consider sex in studies investigating health impacts ensuing from the ingestion of functional foods or ingredients aimed at improving gut health.

## Abbreviations

BCFA: branch-chain fatty acid
BUN: blood urea nitrogen
CD: cluster of differentiation
CINC-1: cytokine-induced chemoattractant-1
dp: degree polymerization
FCRα1: fragment, crystallizable receptor α1
FOS: fructooligosaccharide
GALT: gut-associated lymphoid tissue
MLN: mesenteric lymph node
OF: oligofructose
T_H_2: T helper 2
TLR: toll-like receptor
